# Circulating tumor cells in lobular versus ductal metastatic breast cancer

**DOI:** 10.1007/s10549-026-08111-3

**Published:** 2026-10-26

**Authors:** Emily M. Dolce, Elizabeth P. Darga, Nicholas C. Henderson, Dafydd G. Thomas, David A. Hanauer, Arul M. Chinnaiyan, Erin F. Cobain, N. Lynn Henry, Costanza Paoletti, Andi K. Cani, Daniel F. Hayes

**Affiliations:** 1 Breast Oncology Section, Department of Internal Medicine, University of Michigan Medical School, CCC 6431, 1500 E. Medical Center Drive, MI Ann Arbor, 48109-5942 USA; 2 University of Michigan Rogel Cancer Center, University of Michigan, Ann Arbor, MI USA; 3 Department of Biostatistics, School of Public Health, University of Michigan, Ann Arbor, MI USA; 4 Department of Pathology and Michigan Center for Translational Pathology, University of Michigan Medical School, Ann Arbor, MI USA; 5 Department of Pediatrics and School of Information, University of Michigan, Ann Arbor, MI USA

**Keywords:** Breast cancer, Invasive lobular breast carcinoma, Circulating tumor cells

## Abstract

**Purpose:**

Infiltrating lobular carcinoma (ILC) accounts for ~ 10% of all invasive breast cancer histologies. ILC is associated with worse prognosis than infiltrating ductal cancer. Circulating tumor cells (CTCs) are commonly elevated in metastatic breast cancer (mBC) and elevated levels of CTCs are associated with worse prognosis in breast cancers. We investigated CTC incidence in ILC in two patient cohorts.

**Methods:**

Data from electronic medical records at the University of Michigan were identified using the EMERSE search engine to assess CTC levels in patients with mBC, using CellSearch^®^. Findings were validated in a separate cohort of mBC patients who participated in a research protocol where CTC were analyzed.

**Results:**

Within the EMERSE cohort of 218, 38 (17%), 29 (13%) and 151 (69%) patients, had lobular, mixed, and ductal mBC subtype, respectively. Overall, 101/218 (46%) patients had elevated CTC (≥ 5/7.5 ml whole blood). In this cohort, patients with ILC were more likely to have elevated CTCs (28/38; 74%) compared to those with mixed (14/29; 48%) or ductal (59/151; 39%) mBC (*p* = 0.001). In multivariate regression, patients with ILC were seven times more likely than ductal mBC to have elevated CTCs. These data were validated in 146 separate mBC patients in the research cohort. Elevated CTCs were detected in 79%, 31%, and 36% of patients with lobular, mixed and ductal mBC, respectively.

**Conclusion:**

CTC are more commonly elevated and tend to be higher in magnitude in patients with lobular compared to ductal mBC. CTC levels in mixed ductal-lobular morphology numerically more resemble ductal mBC.

## Introduction

95% of all invasive breast cancers are of either ductal (~ 80%), lobular (~ 10%) or mixed ductal/lobular (~ 5%) histology [1, 2]. Invasive lobular cancer (ILC) is almost always estrogen receptor (ER) positive and HER2 negative [1, 3]. For decades, most studies suggested that ER positive ILC and invasive ductal cancer (IDC) have the same prognosis and they have been essentially treated identically. Recently, several studies have suggested that, despite its favorable hormone receptor profile, early-stage ILC is associated with less favorable long-term outcome, stage for stage, than IDC [4]. In addition, ILC appears to be less sensitive to chemotherapy as reflected by lower pathologic complete response rate following neoadjuvant treatment [5]. Although ILC is highly endocrine responsive, emerging evidence suggests differential sensitivity to specific endocrine agents, with aromatase inhibitors providing greater benefit than tamoxifen in this histologic subtype [2, 3, 6–12]. Regardless, ILC clearly differs from IDC in terms of metastatic patterns. ILC tends to metastasize to unusual locations, such as mesothelial and intra-luminal surfaces, suggesting it has a unique metastatic biology [13–15].

Liquid biopsies, such as circulating tumor cells (CTCs), overcome the challenges inherent in tissue biopsies for serial monitoring of cancer biomarkers [16]. The CellSearch^®^ system, based on anti-EpCAM CTC capture, is the most widely used assay to identify, enumerate, and characterize CTCs. CTC levels using this assay are highly prognostic in metastatic breast (mBC) and other cancers [16–18]. Prior studies have suggested that patients with lobular compared to ductal mBC have a significantly higher incidence of CTCs as well as higher total CTC counts compared to, suggesting a more aggressive biology and worse clinical outcome [19–23].

We leveraged two mBC data sets in our institution [24, 25] to further explore the real-world evidence for incidence of elevated levels, including absolute numbers, of CTCs in metastatic ILC, mixed, and IDC.

## Methods

### EMERSE patient clinical cohort and electronic medical record abstraction

To develop a test set, we conducted a retrospective electronic medical record chart review study. Real-world clinical data from electronic medical records at Michigan Medicine were identified using the EMERSE medical record search engine [24]. CTC levels, as enumerated using the CellSearch^®^ system, were assessed in patients with metastatic breast cancer. After obtaining approval from the Institutional Review Board (IRB) at the University of Michigan Medical School (HUM00173456; Clinical trial number: not applicable; Human Ethics and Consent to Participate declarations: not applicable), we used the EMERSE tool to identify a starting patient population treated at the University of Michigan (UM) who fit the following search terms criteria: Breast AND (CTC OR “circulating tumor cell” OR “circulating tumor cells” OR “CellSearch” OR “CTCs”). Medical charts from identified patients were then reviewed to ascertain the mention of metastatic breast cancer and circulating tumor cell results that were determined using the commercially available CellSearch^®^ system. This population of patients was subjected to a final screen to ensure they met the following eligibility criteria: Female, mBC, pathology information or archived tissue available to determine breast cancer histology, and clinical CellSearch^®^ CTC results.

For the patients who met the eligibility criteria, we then performed an abstraction of the electronic medical record for other relevant clinical data, including date of breast cancer diagnosis and metastasis, stage/grade/TNM information at diagnosis, date and result of CellSearch^®^ CTC test, sites of disease, current (at time of CTC evaluation) and previous lines of therapy, and treatment status. Tumor pathology information including ER, progesterone receptor (PgR), human epidermal growth factor 2 (HER2) was obtained, and histology of the primary tumor (lobular/mixed/ductal) was recorded, unless unavailable, in which case, the histological designation of the metastatic tissue was used. Patients with histologies other than ILC, IDC, and mixed ILC/IDC were excluded. Tissue histology types were based solely on pathologic tissue review, and not on molecular features (such as E-cadherin loss), using standard histopathologic criteria [26].

Patients whose tumors could not be classified as ILC, IDC, or mixed from chart abstraction were further investigated to determine if they had archived primary or metastatic tissue at UM to re-evaluate for ILC vs. IDC vs. mixed status. We retrieved archived tissue collected from these patients, and the H&E slides were re-reviewed if available or, if not, the tissue was re-stained to evaluate for ILC vs. IDC vs. mixed status. Additionally, all cases classified as ILC that were not ER positive and HER2 negative were re-reviewed and re-stained for ER and HER2 at UM.

All CTC evaluations for the Clinical Cohort were performed in a clinical pathology laboratory according to the manufacturer’s instructions. Patients with CTCs drawn before starting first line treatment of mBC of any type and who had CTCs drawn within a 1-week window before or after starting treatment were included. Likewise, patients with progression on their most recent line of metastatic therapy and who went on to receive second or later line of therapy were also included in the final population if they had not previously been included in this study and if they had CTCs drawn within a 1-week window of progression on their most recent line of metastatic therapy. We chose a 1-week window based on previous reports that showed no major difference in CTC incidence between baseline and 72 h, but a decrease in CTC incidence by day 15 or first follow up [27, 28]. If more than one time point fit our criteria, the time point closest to the start of the patient’s next line of therapy was chosen for inclusion in the analysis.

### MI-ONCOSEQ patient research cohort

The MI-ONCOSEQ Protocol (HUM00046018) was conducted at the UM Rogel Cancer Center to perform comprehensive next generation sequencing of metastatic tissue biopsies collected from patients with any type of metastatic solid tumors [25]. Patients with mBC (*n* = 146), who were not part of the EMERSE Clinical Cohort, and who were enrolled in the MI-ONCOSEQ Protocol were also enrolled in a companion protocol (MI-CTC-ONCOSEQ Protocol, HUM00075395; Clinical trial number: not applicable) [29], after signing separate informed consent to participate approved by the UM IRB and in accordance with the Declaration of Helsinki. The MI-CTC-ONCOSEQ protocol allowed blood collection for cell free tumor DNA and CTCs. Blood collection, processing, and CTC enrichment and purification methods were performed in an investigational, non-clinical laboratory at UM as previously described [17, 30]. Briefly, blood collected into three separate CellSave tubes (Menarini Silicon Biosystems S.p.A., Bologna, Italy) was pooled, mixed, re-aliquoted to 7.5 mL, and processed for CTC enrichment using the CellSearch^®^ CTC enrichment system with the CXC Kit (Menarini Silicon Biosystems) [31, 32]. Chart review was conducted to determine the breast cancer histology, ER, PgR, and HER2 status for these patients.

### Statistical analyses

These data are reported according to the REMARK criteria [33].

All statistical analyses were exploratory and hypothesis generating to better understand the role of CTCs in ILC. Descriptive statistics including the mean, standard deviation, median, and range of CTC counts were calculated for the entire cohort and for the ILC, IDC, and mixed lobular and ductal subgroups. We explored the relationship between the CTC count and histopathologic type of breast cancer, adjusting for the number of sites of disease, presence of bone versus visceral metastases, and prior lines of therapy, using logistic regression. Overall survival among mBC patients was evaluated using the Kaplan-Meier method, and a comparison of overall survival between lobular and ductal mBC patients was performed using a log-rank test. All analyses used pathology information from patients’ metastatic tissues, when available. In cases where the metastatic tissue information was unavailable, primary tissue information was used. The fractions of patients with ≥ 5 CTC / 7.5mL whole blood (WB) were compared across subtypes using a chi-squared test.

## Results

### Clinical cohort patient population

**Fig. 1:**
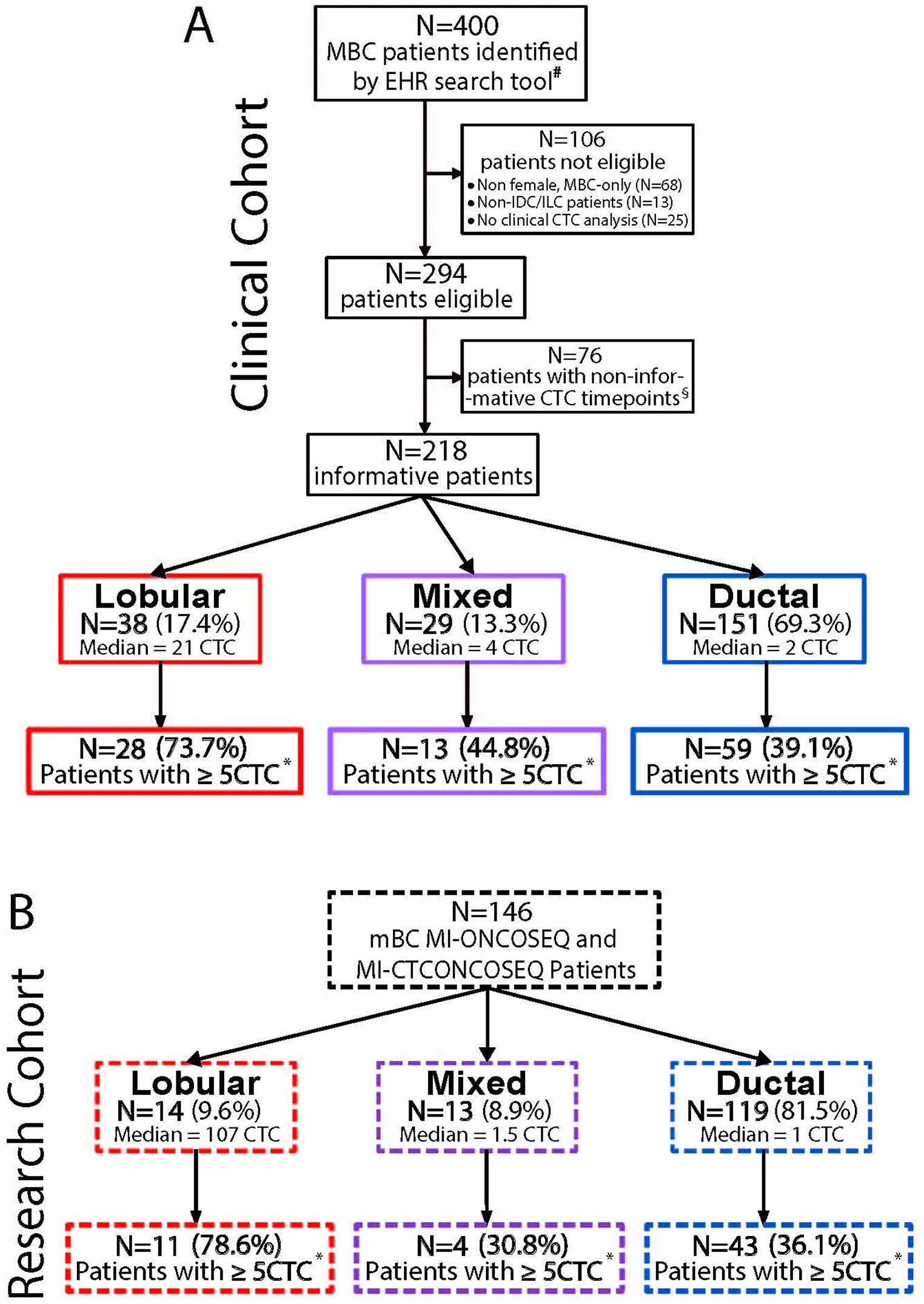
REMARK diagram of patients and circulating tumor cell enumeration in metastatic breast cancer by histological subtype (See Methods for details) **a**) ^#^EMERSE (Electronic Medical Record Search Engine) Clinical Cohort **b**) Mi-CTC-ONCOSEQ Research Cohort § Informative CTC timepoint = CTC assessment at baseline before 1st line or within one week of progression on any line of treatment in the metastatic setting. * Per 7.5mL whole blood

A search of the UM electronic health records using the EMERSE tool [24] identified 400 patients with mBC who had undergone clinical CTC assessment using the CellSearch^®^ platform within a 14-year period (August 2005-June 2019). Of these, 294 (74%) patients met inclusion criteria (designated the Clinical Cohort), of whom 218 (55%) had CTC results at informative timepoints as priorly defined (Fig. 1A; Table 1).

**Table 1:** Clinical cohort patient characteristics

	Lobular (*N* = 38, 17%)	Mixed (*N* = 29, 13%)	Ductal (*N* = 151, 69%)	Overall (*N* = 218)
Primary ER Status				
Positive	35 (92.1%)	23 (79.3%)	106 (70.2%)	164 (75.2%)
Missing	2	0	9	11
Primary PgR Status				
Positive	25 (65.8%)	22 (75.9%)	82 (54.3%)	129 (59.2%)
Missing	3	0	14	17
Primary HER2 Status				
Positive	0 (0%)	5 (17.2%)	29 (19.2%)	34 (15.6%)
Missing	6	3	24	33
Site of Disease				
Bone (only or with other sites)	31 (81.6%)	24 (82.8%)	99 (65.6%)	154 (70.6%)
Liver	7 (18.4%)	13 (44.8%)	58 (38.4%)	78 (35.7%)
Num Sites of Disease Mean (SD)	1.68 (0.933)	2.85 (1.38)	2.40 (1.31)	2.29 (1.29)
Met Num Lines Prior ET				
0	28 (73.7%)	20 (69.0%)	111 (73.5%)	159 (72.9%)
1	7 (18.4%)	6 (20.7%)	19 (12.6%)	32 (14.7%)
2	1 (2.6%)	1 (3.4%)	11 (7.3%)	13 (6.0%)
≥ 3	2 (5.3%)	2 (6.9%)	10 (6.6%)	14 (6.4%)

(ER=Estrogen Receptor, PgR=Progesterone Receptor, HER2 = Human Epidermal Growth Factor Receptor 2, SD=standard deviation)

In this cohort, 38 (17%), 29 (13%) and 151 (69%) patients had lobular, mixed ductal-lobular, and ductal mBC subtypes, respectively. As expected, of patients with informative IHC results, 35/38 (92%) patients with lobular mBC vs. 23/29 (79%) and 106/151 (70%) with mixed or ductal mBC, respectively, had ER-positive cancers (Table 1). No patients with lobular mBC vs. 5 (17%) and 29 (19%) of patients with mixed or ductal mBC, respectively, had tumors that were positive for HER2, either by immunohistochemistry or in situ hybridization. In this cohort, 31 (82%) and 7 (18%) of patients with lobular mBC had bone and/or liver metastases, respectively, at the relevant time of CTC evaluation. Patients with mixed mBC had 24 (83%) and 13 (45%) bone and/or liver metastases, respectively, and those with ductal mBC had 99 (66%) and 58 (38%) had disease in bone and/or liver, respectively.

**Fig. 2:**
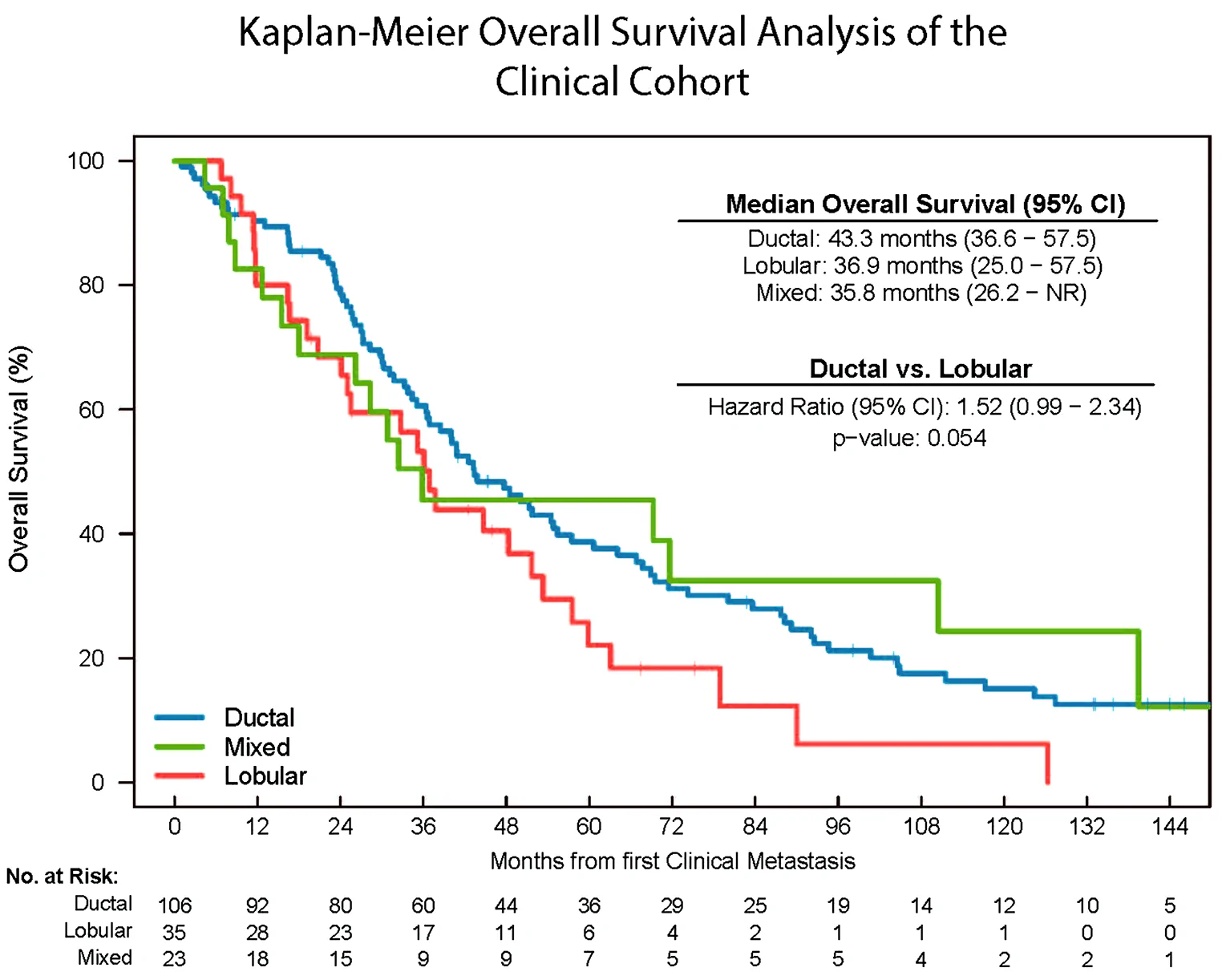
Overall survival from time of first metastases in ER positive patients with lobular, mixed, or ductal metastatic breast cancer in clinical cohort (see methods for details)

With a median follow-up of 136 months, patients with ER-positive lobular mBC had a median overall survival from the time of first metastasis of 36.9 months (95% Confidence Interval (CI) 25.0–57.5). This was numerically shorter than the median overall survival among ductal mBC patients of 43.3 months (95% Confidence Interval (CI) 36.6–57.5), with this difference being nearly statistically significant (Hazard Ratio (HR) 1.52, 95% Confidence Interval (CI) 0.99–2.34, *p* = 0.054, Fig. 2).

### CTC enumeration in patients with metastatic lobular breast cancer

Overall, 101/218 (46%) patients in the Clinical Cohort had elevated CTC (≥ 5/7.5 ml WB) at baseline prior to initiation of the relevant line of therapy (Fig. 1A), with the distribution of CTC across the spectrum above 5/7.5 ml WB as shown in Table 2. Patients with lobular mBC were more likely to have elevated CTCs (28/38; 74%, median 24 cells/patient) compared to those with mixed (14/29; 48%, median 4 cells/patient) or ductal (59/151; 39%, median 2 cells/patient) mBC (Fig. 1A; Table 2; lobular mBC vs. mixed or ductal *p* = 0.001). Moreover, patients with lobular mBC were also more likely to have very high levels of CTC. One-half of patients with lobular mBC compared to 24% and 17% of patients with mixed or ductal mBC had ≥ 26 CTC/7.5 ml WB (Table 2; lobular mBC vs. mixed or ductal, Pearson chi-square test *p* < 0.001). Indeed, among patients with lobular mBC that had elevated CTCs, they were found at a median of 32 cells/patient compared to 25 and 24 cells/patient in patients with mixed and ductal mBC, respectively (data not shown).

**Table 2:** Clinical cohort CTC count distribution by subtype

CTC Count (7.5mL WB)	Lobular (%) *N* = 38	Mixed (%) *N* = 29	Ductal (%) *N* = 151	Overall *N* = 218
< 5	10 (26.3%)	15 (51.7%)	92 (60.9%)	117 (53.7%)
≥ 5	28 (73.7%)	14 (48.3%)	59 (39.1%)	101 (46.3%)
5–10	5 (13.2%)	4 (13.8%)	16 (10.6%)	25 (11.5%)
11–25	4 (10.5%)	3 (10.3%)	17 (11.3%)	24 (11.0%)
≥ 26	19 (50.0%)	7 (24.1%)	26 (17.2%)	52 (23.9%)

In multivariate regression analysis, patients with lobular mBC had an odds of having elevated CTCs that was more than seven times larger than those with ductal mBC (Table 3). Sites of disease were also statistically associated with elevated CTCs, but not to the same degree as histologic subtype.

**Table 3:** Multivariate logistic regression analysis of elevated CTC counts

Variable	Odds Ratio (95% CI) of having ≥ 5 CTC/7.5 ml WB	*p*-value
Histologic Type		
Ductal	Ref.	
Lobular	7.3 (3.0–17.9)	< 0.001
Mixed	1.2 (0.5–2.9)	0.71
Site of Disease		
Bone (yes vs. no)	3.3 (1.6–7.1)	0.002
Lymph Node (yes vs. no)	3.3 (1.4–7.5)	0.005
Liver (yes vs. no)	4.1 (1.9–8.6)	< 0.001
Number of Sites of Disease (≥ 3 sites vs. < 3 sites)	1.0 (0.4–2.2)	0.93
Number of Prior Lines of Metastatic Therapy (≥ 1 prior lines vs. 0 prior lines)	0.7 (0.3–1.3)	0.23

**Fig. 3:**
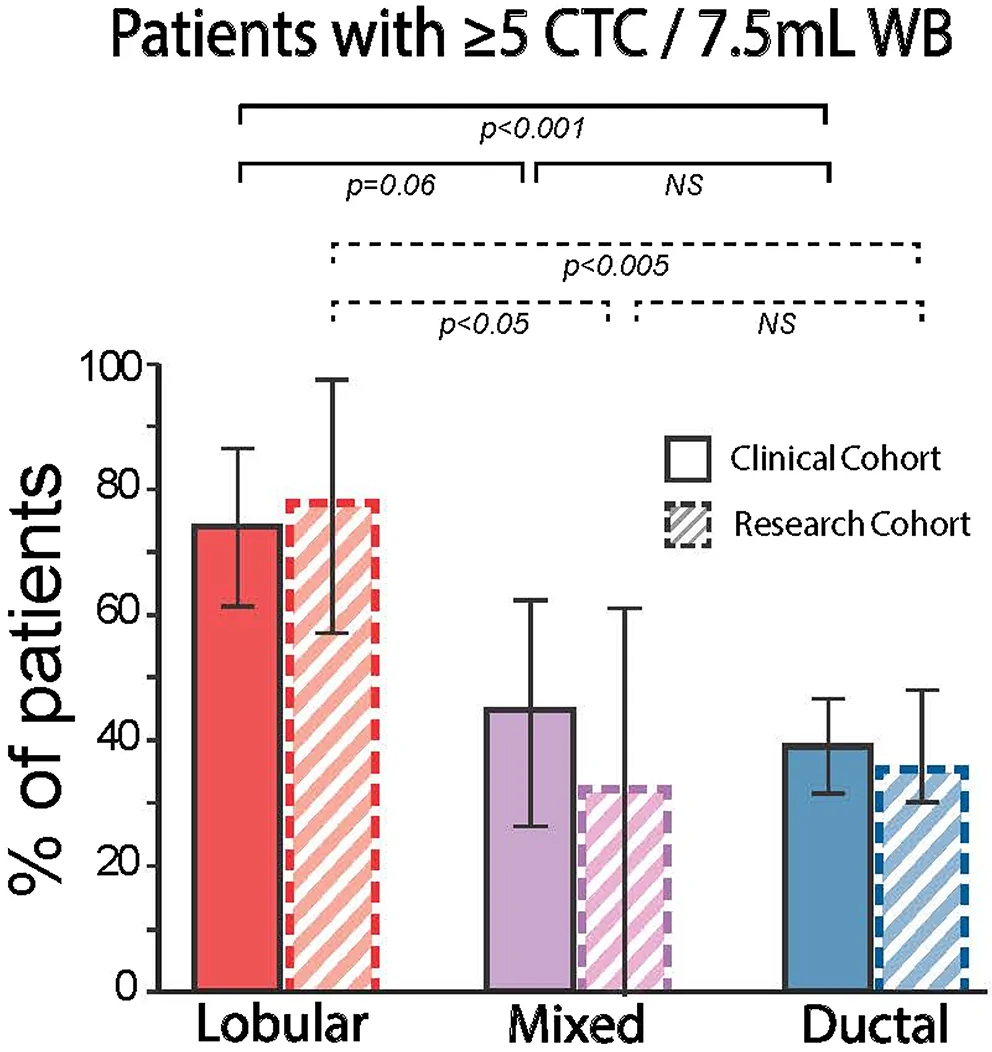
Percentage of patients with ≥ 5 CTC/7.5mL whole blood for clinical (filled) and research (hatched) cohorts error bars: 95% CI; *p* values: two-proportion Z-Test; NS: *p* > 0.05

To verify these findings, we analyzed a second group of patients, designated the Research Cohort, of 146 patients with mBC who were enrolled in the MI-CTC-ONCOSEQ program. (Fig. 1B). CTC counts among the three histologic subtypes in this cohort were similar to those seen in the clinical cohort. Among the 14 patients with lobular histology, 11 (79%) had ≥ 5 CTC/7.5 ml WB at their baseline draw compared to 4 of 13 (31%, *p* = 0.02) and 43 of 119 (36%, *p* = 0.002) patients with mixed or ductal mBC, respectively (Figs. 1B and 3).

## Discussion

In this study, we have observed in two separate cohorts that patients with lobular compared to ductal mBC are more likely to have elevated CTCs (≥ 5CTC/7.5 ml WB): 75–80% compared to 35–40%. Furthermore, patients with lobular compared to ductal mBC also appear to have higher absolute CTC levels if they are elevated above 5 CTC/7.5 ml WB, with a median of 32 vs. 24 cells/patient.

Our findings confirm previously reported data from some, but not all, prior studies that patients with lobular compared to mixed or ductal mBC are substantially more likely to have elevated CTC counts [19–23, 34]. Although all patients were treated in a single institution, we believe that our data are likely to be representative of the broader mBC population as a whole, since they were observed in both a real-world clinical cohort and validated in a prospective research cohort. Furthermore, the fraction of patients with ductal mBC with elevated CTC, 35–40%, is consistent with many prior studies of CTCs in breast cancer, most of which were dominated by IDC [18].

In the Clinical Cohort, we observed that overall survival was shorter for patients with lobular vs. ductal mBC. This result is consistent with prior reports suggesting that lobular carcinoma carries a slightly worse prognosis when compared to equivalent cohorts of ductal cancer, matched by stage and hormone receptor status [35]. We speculate that this worse OS is a biologic function of higher rates of CTCs and therefore tumor dissemination. Indeed, elevated CTCs have consistently been associated with worse prognosis in both early and advanced/metastatic breast and other solid malignancies [16, 18, 36].

However, in addition to CTCs, circulating cell free tumor DNA (ctDNA) is a form of liquid biopsy that might be valuable in evaluation and monitoring of breast and other cancers [37]. As with CTCs, elevated ctDNA levels are also associated with worse prognosis in mBC [38]. A few scattered studies have evaluated the presence of ctDNA in patients with lobular mBC [35, 39–42]. In these reports, ctDNA and CTC incidence were not compared directly, regardless of histologic subtype, but ctDNA appears to be more commonly detected than CTCs in patients with mBC. Although the CTC and ctDNA genetic results in these studies depended on the specific assay used, they basically mimic those reported in much larger numbers for patients with ductal mBC [35, 40].

Beyond enumeration of CTCs or quantification of ctDNA, further characterization could be important to predict response to specific, targeted therapies and to evaluate emergence of tumor heterogeneity and clonal evolution under therapeutic pressure. Although CTCs may not be elevated as frequently as ctDNA, they have the advantage of offering phenotypic analyses. We and others have reported baseline and serial CTC expression of a variety of relevant tumor biomarkers, including ER, HER2, Bcl2, Ki67, M30 (a marker of apoptosis), and programmed death ligand (PD-L1) [28, 43, 44].

Moreover, CTC genomics may complement that of ctDNA [29, 45–48]. Genetic analyses of ctDNA have demonstrated similarity between lobular and ductal cancers, except for the presence of abnormalities in CDH1, the gene that encodes for E-cadherin, a hallmark finding in lobular cancer [3, 35, 40, 49, 50]. Of interest, in tissue studies, mutations in the *ESR1* gene, which encodes for ER, are related to resistance to estrogen depletion strategies, such as treatment with aromatase inhibitors [51]. These mutations can be identified in ctDNA and detection of their emergence has been used to guide therapy towards selective estrogen receptor downregulation [52, 53]. However, Desmedt et al. have reported the tissue frequency of *ESR1* mutations by droplet digital PCR is approximately 9% and that there are no differences in prevalence and distribution of the mutations between lobular and ductal mBC [54]. Relevant to our study, they observed that *ESR1* mutations were detected in ctDNA in 20 of 76 (26.3%) patients with ER-positive lobular, compared to 123/416 (29.6%) patients with ductal, mBC (*p* = 0.68). By contrast, Davis et al. reported that single nucleotide variations were higher in lobular than ductal mBC, as were mutations in ERBB2 (OR 3.6, [95% confidence interval (CI) 1.6–8.2]), and *PTEN* (OR 2.5, [95% CI 1.05–5.8]) [40]. 

The emergence of phenotypic and genotypic clonal heterogeneity during treatment might provide insight into informed personalized combination therapies rather than the general empiric strategies used currently. Although at present no studies have demonstrated absolute clinical utility of CTCs vs. ctDNA molecular characterization, we anticipate that ongoing clinical trials should provide further insight into this exciting use context of liquid biopsies.

Our study is strengthened by the observation that the real-world evidence Clinical Cohort is consistent with the known characteristics of breast cancer in general in regard to hormone receptor and HER2 status, as well as sites of disease. Further, in the Clinical Cohort, patients with ER positive lobular compared to ER positive ductal mBC had a worse overall survival from time of diagnosis of their metastatic disease (Fig. 2).

Nonetheless, both cohorts suffer from patient selection. In the Clinical Cohort, the decisions of when, and from what patients, CTCs were drawn were at the clinicians’ discretion. Likewise, Research Cohort patient enrollment to the MI-ONCOSEQ and therefore the MI-CTC-ONCOSEQ protocols was based on their clinical circumstances and physicians’ decisions. We did not analyze outcomes (such as progression free or overall survival) according to CTC levels because of the confounding variability of when the CTCs were drawn for each patient (first, second, third lines), the heterogeneity of intrinsic subtypes, and the lack of prospective proscription for standardized follow-up type, timing, and reading of scans. Also, given the retrospective nature of this study, we were unable to accurately determine specific sites of metastases other than the major ones (bone, liver) and therefore were not able to investigate the widely recognized curious metastatic pattern associated with lobular cancers [13–15, 55–57]. Other authors have also suggested that ER positive lobular cancer may be less sensitive to endocrine therapy compared to ductal cancer, but we were unable to interrogate this issue, as well [58].

In summary, CTC levels are more commonly elevated in patients with lobular compared to ductal mBC, and these elevations tend to be of greater magnitude than those observed in patients with ductal mBC. Future studies should extend beyond CTC enumeration to include comprehensive molecular characterization, such as phenotypic assessment of biomarker expression and genotyping for cancer-related genetic abnormalities. In addition, further research is needed to determine the clinical significance of emergence of distinct, therapeutically targetable clones during treatment.

## Data Availability

The datasets generated during and analyzed during the current study are not publicly available but are available from the corresponding author on reasonable request.
